# Phytochemical, Antioxidant and Anti-Cancer Properties of *Euphorbia tirucalli* Methanolic and Aqueous Extracts

**DOI:** 10.3390/antiox4040647

**Published:** 2015-10-08

**Authors:** Benjamin Munro, Quan V. Vuong, Anita C. Chalmers, Chloe D. Goldsmith, Michael C. Bowyer, Christopher J. Scarlett

**Affiliations:** 1 Pancreatic Cancer Research, Nutrition Food & Health Research Group, University of Newcastle, Ourimbah, NSW 2258 Australia; E-Mails: benjamin.munro@uon.edu.au (B.M.); vanquan.vuong@newcastle.edu.au (Q.V.V.); chloe.d.goldsmith@uon.edu.au (C.D.G.); michael.bowyer@newcastle.edu.au (M.C.B.); 2 School of Environmental and Life Sciences, University of Newcastle, Ourimbah, NSW 2258, Australia; E-Mail: anita.chalmers@newcastle.edu.au; 3 Cancer Research Program, Garvan Institute of Medical Research, Darlinghurst, NSW, 2010, Australia

**Keywords:** *Euphorbia tirucalli*, polyphenols, antioxidant, pancreatic cancer

## Abstract

*Euphorbia tirucalli* is a succulent shrub or small tree that is native to the African continent, however, it is widely cultivated across the globe due to its use in traditional medicines to treat ailments, ranging from scorpion stings to HIV. Recent studies have identified compounds present in the latex of the plant, including a range of bi- and triterpenoids that exhibit bioactivity, including anticancer activity. This study aimed to optimize water extraction conditions for high-yield total phenolic content recovery, to prepare methanol and aqueous extracts from the aerial sections of the plant, and to test the phytochemical, antioxidant, and anti-cancer properties of these extracts. Water extraction of total phenolic compounds (TPC) was optimized across a range of parameters including temperature, extraction time, and plant mass-to-solvent ratio. The water extract of the *E. tirucalli* powder was found to contain TPC of 34.01 mg GAE (gallic acid equivalents)/g, which was approximately half that of the methanol extract (77.33 mg GAE/g). The results of antioxidant assays showed a uniform trend, with the methanol extract’s antioxidant reducing activity exceeding that of water extracts, typically by a factor of 2:1. Regression analysis of the antioxidant assays showed the strongest correlation between extract TPC and antioxidant activity for the ABTS (2,2-azino-bis(3-ethyl-benzothiazoline-6-sulfonic acid) and DPPH (2,2-diphenyl-1-picrylhydrazyl) methods. The methanol extract also showed greater growth inhibition capacity towards the MiaPaCa-2 pancreatic cancer cell line. These data suggest that further investigations are required to confirm the source of activity within the *E. tirucalli* leaf and stems for potential use in the nutraceutical and pharmaceutical industries.

## 1. Introduction

The historical use of *E.* *tirucalli* (family *Euphorbiaceae*) in traditional medicine in the Middle East, India, Africa, and South America was to treat a range of ailments, including syphilis, asthma, cancer, colic, intestinal parasites, skin diseases, and leprosy [1,2,3]. Consequently, this has prompted scientific interest in its pharmacological properties. Chromatographic and spectroscopic analysis of extracts from the photosynthetic stems have identified a range of phenolics and terpenes, the most prominent of which are the triterpenes euphol and tirucallol [4,5]. Leaf/stem extracts have been shown to possess potent antioxidant properties [6,7], a key factor in combating cellular oxidative stress [8]. Previous studies have linked *E. tirucalli* whole plant methanol extracts with positive antioxidant activity, potentially due to their high phenolic content, and have been deemed an excellent and accessible source of natural antioxidant activity [9,10].

The use of *E. tirucalli* latex in traditional medicine as a treatment for cancer has attracted the recent interest of the West [2]. However, this must be treated with caution, as whole plant aqueous extracts have been shown to interact with antioxidant enzyme systems in human leukocytes via upregulation of key antioxidant enzyme genes. This leads to increased cytoxocity, confirming the need for precise investigations into dose and administration of *E. tirucalli* extracts for medicinal purposes [11]. A further study [12] assessed the anticancer properties of euphol extracted from *E. tirucalli* latex, finding it to exhibit dose and time dependent cytotoxic effects against a significant number of cell lines, with most prominent effects against oesophageal squamous cell and pancreatic cell carcinomas.

The ferulic acid and antioxidant properties of *E. tirucalli* have been reported in a previous study [7]. Other studies have also extracted bioactive compounds from *E.* *tirucalli* [5,11,13], however, investigation of the optimal extraction conditions of phenolic compounds from *E.* *tirucalli* have focused on the use of organic solvents for extraction [13], or on the extraction of specific compounds [5].

Water was selected for this study as it is safe, environmentally friendly, accessible, and cheap in comparison with the organic solvents utilised in previous studies [13]. According to the U.S. Food and Drug Administration (FDA), methanol is also considered as a safe solvent for the extraction of bioactive compounds [14]. As such, this study aimed to optimize water extraction conditions for high yield total phenolic content recovery, and to prepare methanol and aqueous extracts from the aerial sections of *E. tirucalli*. The phytochemical properties and the antioxidant capacity of the aqueous and methanolic extracts were assessed and compared using four different antioxidant assays, while the efficacy of these aqueous and methanolic extracts in limiting the growth of pancreatic cancer cells was also evaluated. These findings can be considered for further isolation and purification of phenolic compounds from *E. tirucalli* for potential use in the nutraceutical or pharmaceutical industries.

## 2. Experimental Section

### 2.1. Specimen Harvesting

The leaves and stems of a *Euphorbia tirucalli* tree were harvested on 16 July 2014, from a property located in Saratoga, New South Wales (NSW), Australia (33.47° S, 151.35° E). The plant was authenticated by one of the authors (A.C.C.) and a voucher specimen deposited at the Don McNair Herbarium, the University of Newcastle, NSW, Australia. The leaves and stems were cut and stored at −20 °C until required. The plant was prepared for extraction by freeze-drying the leaves and stems, then grinding the dried material to a fine powder in a blender. All samples were stored at −20 °C prior to extraction.

### 2.2. Methanol Extraction

**Figure 1:**
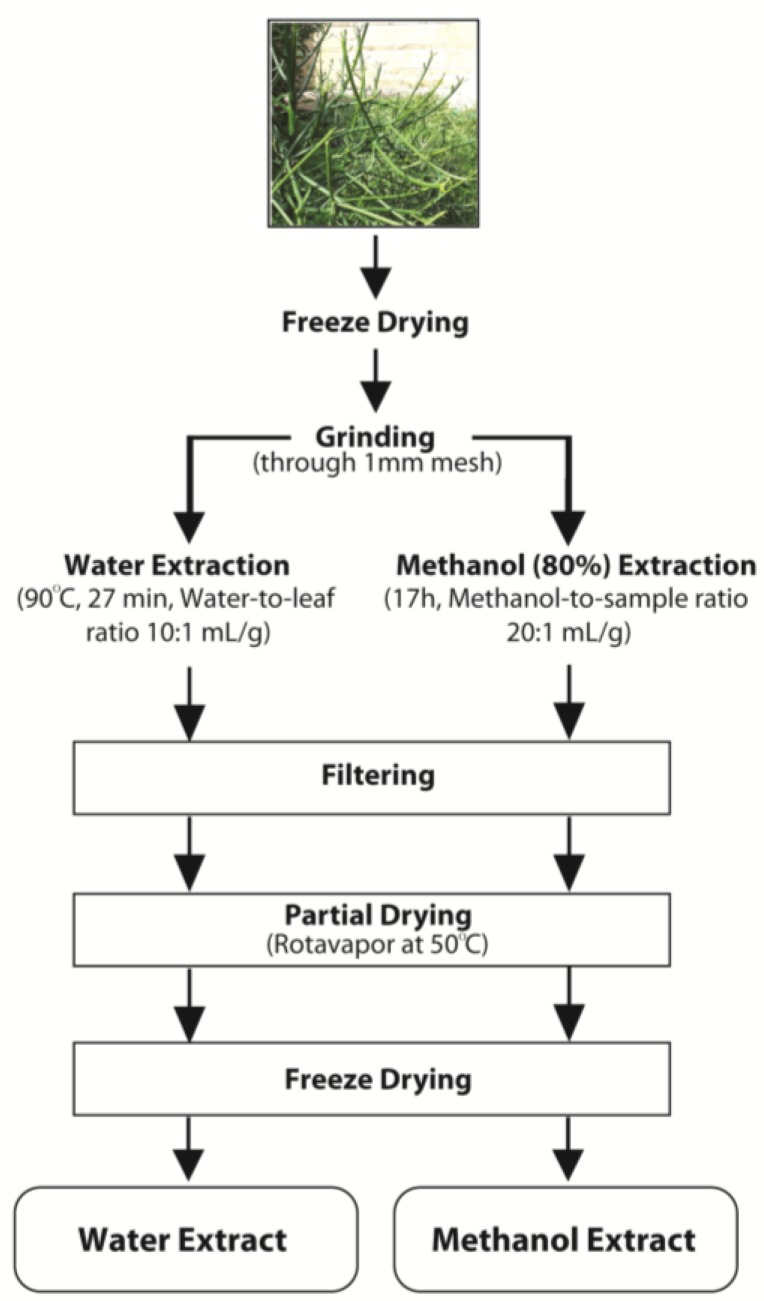
Schematic diagram for the extraction process for water and methanol extracts.

The extraction process is outlined in Figure 1. Freeze-dried, ground whole plant material (20.0 g) was extracted by stirring in 400 mL of 80% (v/v) methanol for 17 h at room temperature (magnetic stirrer), followed by sonication (2 h) in an ultrasound bath at 150 W (Soniclean 1000HD, Thebarton, South Australia, Australia). The extract was then filtered under reduced pressure (Advantec 90 mm, Caringbah, NSW, Australia) to separate the insoluble plant material and the solvent evaporated at 50 °C under reduced pressure in a rotary evaporator (Buchi Rotavapor B-480, Buchi Australia, Noble Park, Australia). The resulting concentrate was then mixed with 15 mL of deionised (DI) water, frozen with liquid nitrogen and freeze-dried to obtain a crude methanol extract powder, which was then stored at −20 °C until required.

### 2.3. Aqueous Extraction

An aqueous extraction method was optimized using Response Surface Methodology (RSM) to examine the impact of experimental parameters, such as extraction time, solvent temperature, and sample-to-solvent ratios on the total phenolic content (TPC) extracted from frozen plant samples.

Numerous experiments were conducted to identify the optimal ranges of temperature and time using one-factor design. For the temperature range, extracts were prepared by crushing 1 g of thawed plant material using a mortar and pestle followed by extraction in 50 mL of water for 15 min. at room temperature and temperatures ranging between 40 °C and 90 °C (10 °C intervals). Total phenolic content (TPC) for each of the extracts was then determined and the optimal temperature range was 70 °C to 90 °C.

To determine the optimal time range, plant material (1.0 g) was crushed with a mortar and pestle and extracted in 50 mL of water at 90 °C at time intervals of between 5 and 30 min (5 min intervals). TPC of each extract was then evaluated and the optimal range of time was 20–30 min. These optimal ranges were used to generate an experimental design to determine optimum water extraction conditions using RSM, specifically a Box-Behnken factorial design with 3 center points (Table 1). The experimental data, obtained for the fifteen experimental runs, were fitted to the second-order polynomial model shown in Equation 1:

(1) $$Y=\beta_{0}+\sum_{i=1}^{k}\beta_{i}X_{i}+\sum_{\begin{array}{l} i=1\\ i<j \end{array}}^{k-1}\sum_{j=2}^{k}\beta_{ij}X_{i}X_{j}+\sum_{i=1}^{k}\beta_{ii}X_{i}^{2}$$

where various $X_{i}$ values are independent variables affecting the responses *Y*; $\beta_{0}$, $\beta_{i}$, $\beta_{ii}$ and $\beta_{ij}$ are the regression coefficients for intercept, linear, quadratic, and interaction terms, respectively; and *k* is the number of variables.

Experiments were conducted for TPC in triplicates for each designed condition according to the generated experimental design (Table 1). The data were then analysed to construct a model of the interaction between the variables and TPC to determine the optimum extraction conditions for phenolics. Optimal extraction conditions were found to be 90 °C, 27 min, plant-to-solvent ratio 1:10 g/mL.

### 2.4. Large-Scale Chemical Analysis of Aqueous Extract

Based on the optimal conditions for extraction of TPC using water generated above, a large-scale extraction was performed by blending thawed plant material (25 g) in 250 mL of pre-heated water (90 °C) and shaking in a water bath for 27 min. The procedure was repeated to produce approximately 3.25 L of extract. The extract was then filtered twice under reduced pressure (Advantec 90 mm). The filtrate was then reduced in volume on a rotary evaporator (50 °C), frozen in liquid nitrogen and freeze dried and the resulting powder stored at −20 °C until required.

**Table 1:** Box-Behnken design and observed responses on total phenolic content (TPC) extraction yield of *E. tirucalli*.

Run	Pattern	A, Temperature (°C)	B, Time (min)	C, Plant-Water-Ratio (mL/g)	Extraction Yield (mg GAE/g) *
1	−, −, 0	70	20	1:55	3.36 ± 0.45
2	−, 0, −	70	25	1:10	2.98 ± 0.43
3	−, 0,+	70	25	1:100	4.14 ± 0.20
4	−, +, 0	70	30	1:55	3.09 ± 0.12
5	0, −, −	80	20	1:10	2.33 ± 0.31
6	0, −, +	80	20	1:100	3.29 ± 0.34
7	0, 0, 0	80	25	1:55	2.94 ± 0.00
8	0, 0, 0	80	25	1:55	3.14 ± 1.23
9	0, 0, 0	80	25	1:55	2.62 ± 0.15
10	0, +, −	80	30	1:10	2.26 ± 0.54
11	0, +, +	80	30	1:100	2.91 ± 0.46
12	+, −, 0	90	20	1:55	3.23 ± 0.41
13	+, 0, −	90	25	1:10	3.56 ± 0.79
14	+, 0, +	90	25	1:100	4.26 ± 0.95
15	+, +, 0	90	30	1:55	3.67 ± 0.76

Pattern −, 0, + are the minimum values, centre points, and maximum values, respectively, of temperature, time and plant-water-ratio in the tested ranges. * The values are mean of triplicate experiments.

### 2.5. Moisture Content and Extraction Yield Determination

The moisture content of the fresh plant was determined by mass difference before and after drying in a vacuum oven (Thermoline, VORD-450D, Thermoline Scientific, Wetherill Park, NSW 2164, Australia). Moisture content was expressed as a percentage of the original sample mass. Extraction yield was calculated by taking 5 mL samples (*n* = 3) of filtered, optimized bulk water extract and evaporating to dryness in a vacuum oven. Extraction yield was expressed as the dry mass of extract recovered per gram of plant fresh weight extracted.

### 2.6. Chemical Analysis

#### 2.6.1. Quantification of Total Phenolic Content (TPC)

Total phenolic content (TPC) was determined using a variation of the Folin-Ciocalteu assay [15]. Plant extracts were diluted with the extraction solvent (water or methanol, respectively) to a concentration of 800 µg/mL. One millilitre (1 mL) of the respective solution was then mixed with 5 mL of 10% (v/v) Folin-Ciocalteu reagent, incubated at room temperature (RT) for 5 min., then mixed with 4 mL of 7.5% (w/v) Na_2_CO_3_ and incubated in the dark at RT for 60 min. Solution absorbance (A) was then measured at 760 nm. Gallic acid was used as a standard and TPC concentration was expressed as mg of gallic acid equivalents per gram of sample (mg GAE/g).

#### 2.6.2. Quantification of Total Flavonoid Content (TFC)

Total flavonoid content (TFC) was determined using a modified aluminium chloride assay method [16]. The water and methanol extracts of plant samples were diluted with water or methanol, respectively, to a concentration of 800 µg/mL. Five hundred microlitres of sample, 2 mL of DI water and 150 µL of 5% Na_2_NO_2_ were mixed and incubated at RT for 6 min. 150 µL of 10% AlCl_3_ was then added and incubated for a further 6 min. Finally, 2 mL of 4% NaOH and 700 µL of DI water were added and the solution incubated in the dark at RT for 15 mins. Solution absorbance was recorded at 510 nm with rutin used as the standard. TFC was reported as mg of rutin equivalents per gram of sample (mg·RE/g of the extract).

#### 2.6.3. Total Proanthocyanidin Content

Total proanthocyanidin content was determined using a modified version of the vanillin-HCl method [17]. The water and methanol plant extracts were diluted in their respective solvents to a concentration of 4 mg/mL. A volume of 0.5 mL of the diluted sample was then combined with 3 mL of 4% (w/v) vanillin in methanol and 1.5 mL of 35% HCl and incubated in the dark at RT for 15 min. Solution absorbance was then recorded by UV/visible spectrophotometer (500 nm). A calibration curve was prepared using catechin as the reference standard, with total proanthocyanidin content values were reported as mg of catechin equivalents per gram (mg CE/g of the extract) of sample.

### 2.7. Antioxidant Activity

Water and methanol extracts were diluted in their respective solvents to an initial concentration of 800 µg/mL. This solution was then further diluted to produce three solutions (400, 200, and 100 µg/mL). All solutions were prepared in triplicate and measured to establish if activity was dose-dependent.

#### 2.7.1. Antioxidant Capacity

A modification of the ABTS (2,2-azino-bis(3-ethyl-benzothiazoline-6-sulfonic acid)) assay method [18] was used to assess antioxidant capacity. Stock solution was prepared by combining equal volumes of 7.4 mM ABTS solution and 2.6 mM K_2_S_2_O_8_ and incubating in the dark at RT for >15 h. A working solution was then prepared by diluted an aliquot of the stock solution approximately 1:60 with methanol so as to obtain an absorbance reading A = 1.1 ± 0.02 OD_734 nm_ by UV/Vis spectrophotometry. The assay was then performed by adding 200 µL of sample to 3.8 mL of ABTS^•+^ working solution, followed by an incubation in the dark at RT for 2 hrs. Absorbance was then measured at 734 nm (referenced against the working solution), with the result expressed as a percentage, which was calculated according to Equation 2.

TAC [%] = (A_Control_ – A_Sample_) × 100 / A_Control_ (2)

#### 2.7.2. Free Radical Scavenging Capacity

The radical scavenging capabilities of the extracts were determined using DPPH (2,2-diphenyl-1-picrylhydrazyl) [15]. A DPPH stock solution (0.024% w/v) was prepared in methanol. A working DPPH solution was then prepared by diluting stock solution (1:40) in methanol so as to achieve an absorbance of A = 1.1 ± 0.02 OD_515 nm_ by UV/Vis spectrophotometry. The assay was then performed by adding 200 µL of sample to 3.8 mL of DPPH working solution and incubating at RT for 3 h. Absorbance was then measured at 515 nm (referenced against the working solution), with the result expressed as a percentage, which was calculated according to the above Equation (2).

#### 2.7.3. Ion Reducing Power

Two assays were used to determine the ion reducing power of the extracts; cupric reducing antioxidant capacity (CUPRAC) and ferric reducing antioxidant power (FRAP).

The CUPRAC assay was performed using a modified method of Apak *et al.* [19]. One millilitre (1 mL) of each of the following three reagents: 10 mM CuCl_2_; 7.5 mM neocuproine; 7.7% (w/v) NH_4_Ac were mixed. A volume of 1.1 mL of sample was then added and the mixture incubated in the dark at RT for 90 min. Solution absorbance was measured at 450 nm (referenced against a reagent control).

The FRAP assay was performed, using a modified method developed by Thaipong *et al.* [18]. The following stock reagents were prepared:

(A) 300 mM acetate buffer: 3.1 g sodium acetate trihydrate; 16 mL glacial acetic acid; diluted to 1 L with DI water.
(B) 10 mM TPTZ: 0.3123 g of 2,4,6-tripyridyl-s-triazine (TPTZ) in 100 mL of 40 mM HCl.
(C) 20 mM ferric chloride: 5.406 g of FeCl_3_.6H_2_O in 1 L of DI water.

A working reagent was made up as required by mixing stock reagents A, B, and C in a 10:1:1 ratio. The solution was then warmed to 37 °C. Two hundred microlitres (200 μL) of diluted sample were mixed with 3.8 mL of working reagent and incubated in the dark at RT for 30 min. Solution absorbance was measured at 593 nm (referenced against a reagent control).

### 2.8. Determination of Pancreatic Cell Viability

*Cell culture:* The human pancreatic cancer cell line Mia-PaCa2 (American Type Culture Collection (ATCC), Manassas, VA, USA) was cultured in Dulbecco’s Modified Eagle’s Medium (DMEM) supplemented with 10% fetal bovine serum (FBS), 2.5% horse serum and l-Glutamine (100 µg/mL) at 37 °C, 5% CO_2_.

*Cell Viability:* Cell viability was determined using the Dojindo Cell Counting Kit-8 (CCK-8: Dojindo Molecular Technologies, INC., Maryland, MD, USA). Cells were seeded into a 96 well plate at 5 × 10^3^ cells per well and allowed to adhere for 24 h. The cells were then treated with concentrations of 200, 100, 50, 25, 12.5, and 6.25 µg/mL of crude *E. tirucalli* extract (MeOH and water) or vehicle control and after 72 h 10 µL of CCK-8 solution was added and incubated at 37 °C for 90 min. The absorbance was measured at 450 nm and cell viability was determined as a percentage of control. All experiments were performed in triplicate.

### 2.9. Statistical Analysis

RSM experimental design and analysis were conducted using JMP software (Version 11, SAS, Cary, NC, USA). The software was also used to establish the model equation, to graph the 3-D plot, 2-D contour of the response, and to predict the optimum values for the three response variables. The Student *t*-test was used when there were only two treatments to compare. The one-way analysis of variance (ANOVA) and the least significant difference (LSD) *post hoc* test were conducted using the SPSS statistical software version 20 (IBM, Armonk, New York, NY, USA). Differences between the mean levels of the components in the different experiments were taken to be statistically significant at *p* < 0.05. Correlation coefficient between TPC and antioxidant capacity was performed using Pearson’s correlation (SPSS).

## 3. Results and Discussion

### 3.1. Optimisation of Water Extraction Conditions for TPC

Response Surface Methodology (RSM) was utilised to optimise the extraction of TPC under aqueous conditions. RSM is a mathematical technique developed to optimise an output variable that is influenced by one or more independent variables. The data gathered from a series of test experiments (runs) are then entered into a second order polynomial, which can then be used to predict output response across a range of conditions. Three variables (temperature, extraction time, plant mass to solvent ratios) were assessed in the current study at three levels in a Box-Behnken experimental design (Table 1).

Data from the Box-Behnken experiment implies that the regression model is a reliable tool to predict a response surface over a range of extraction conditions (Table 2; *R*^2^ = 0.956, *p* < 0.01). Analysis of interrelationships between the variables revealed significance between TPC levels extracted and the plant mass to solvent ratio within the range examined. Temperature and extraction time across the range examined did not influence TPC yield significantly.

**Table 2:** Analysis of variance for determination of model fitting.

Source	Degree of Freedom	F-Ratio	Probability > F
Temperature (70, 90)	1	4.0854	0.0992
Time (20, 30)	1	0.2366	0.6473
Ratio (10, 100)	1	37.0704	0.0017 *
Temperature * Time	1	3.1425	0.1365
Temperature * Ratio	1	1.3408	0.2992
Time * Ratio	1	0.6295	0.4635
Temperature * Temperture	1	49.4217	0.0009 *
Time * Time	1	8.2014	0.0352 *
Ratio * Ratio	1	0.8846	0.3901
*R*^2^			0.955754
*p* of lack of fit			0.0069 *

* Significant at *p* < 0.05.

**Figure 2:**
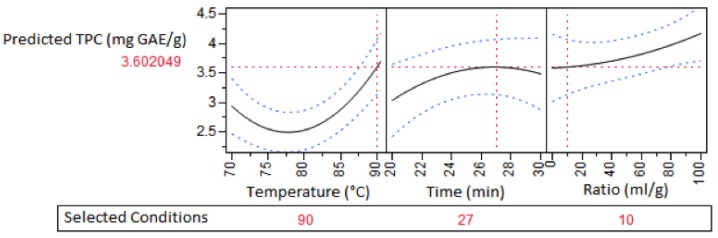
Impact of extraction conditions on predicted total phenolic content (TPC) of dried *E. tirucalli*.

Optimum conditions for maximum TPC from the generated modelling output (Figure 2) were predicted to be temperature = 90 °C, time = 27 min and a plant-to-water ratio = 1:100, with the conditions resulting in a predicted TPC yield of 4.17 mg GAE/g. The high dilution conditions were predicted to be optimal, however considered to be inefficient when time and energy considerations in handling large solvent volumes were taken into consideration. A plant-to-water ratio of 1:10 was therefore chosen for validation, with a predicted TPC yield of 3.60 mg GAE/g, a satisfactory 86% of the theoretical maximum.

In order to validate the modelling predictions, a small-scale experiment was conducted (*n* = 3) using the selected parameters. TPC yield (Table 3) was found to be within the statistical tolerances of the predictive model.

**Table 3:** Validation of TPC yield under optimal extraction conditions predicted by Response Surface Methodology (RSM).

Optimal Extraction Conditions			Predicted Value (mg GAE/g)	Experimental Value (mg GAE/g)
Temperature (°C)	Time (min)	Ratio (g/mL)		
90	27	1:10	3.60 ± 0.47 *	3.43 ± 0.05 *

* Not significant different with *p* > 0.05.

### 3.2. Phytochemical Properties of the E. tirucalli Leaf and Stem Extracts

To date, there has only been one report detailing the antioxidant properties of *E.* *tirucalli* [6]. We therefore undertook a formal evaluation of the physicochemical properties of the plant (Table 4). Moisture content of the freshly harvested plant samples was found to be 78.52%, while the yield of solids from aqueous extraction of fresh plant material was approximately 7%.

The extraction yields of phenolics, flavonoids and proanthocyanidins from freeze-dried plant material was found to be uniformly higher using methanol as a solvent compared to water, with yields in most cases approximately double in comparison (Table 4). Conversely, flavonoid content as a proportion of TPC was found to be slightly higher (42%) in the aqueous extract compared to methanol (37%), with proanthocyanidins extracted in similar amounts in both solvents (2.3% and 2.7%, respectively). TPC compares with analyses performed on fruit, such as guava (170–344 mg GAE/100 g of fresh fruit) [18].

### 3.3. Antioxidant Activity

#### 3.3.1. Antioxidant capacity

**Figure 3:**
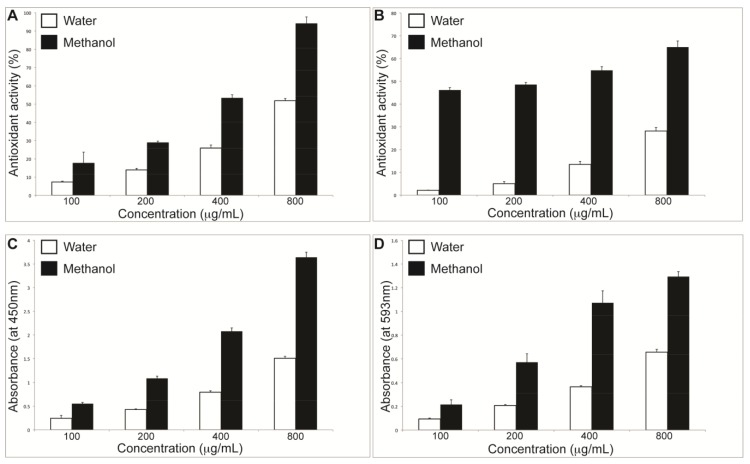
Antioxidant activity of MeOH and water extracts assessed by ABTS (**A**); DPPH (**B**); CUPRAC (**C**); and FRAP (**D**) assays. ABTS: (2,2-azino-bis (3-ethyl-benzothiazoline-6-sulfonic acid); DPPH: (2,2-diphenyl-1-picrylhydrazyl); CUPRAC: cupric reducing antioxidant capacity; FRAP: ferric reducing antioxidant power.

Antioxidant capacity was examined using the ABTS assay and expressed as a percentage (Figure 3A). The methanol extract showed approximately double the activity of the aqueous extract across the concentration range examined, a figure in line with the relative TPC levels reported in Table 4. Antioxidant activity of both extracts was seen to rise with concentration increase, indicating a dose dependent relationship.

**Table 4:** Phytochemical properties of *E. tirucalli* extract.

Property	Sample	Value
Moisture content	Fresh plant	78.52 ± 0.36 (%)
Extraction yield	Water extract	71.7 ± 6.37 (mg/g)
Total phenolic content	Water extract	34.01 ± 1.23 (mg GAE/g)
	Methanol extract	73.33 ± 6.39 (mg GAE/g)
Flavonoids	Water extract	14.24 ± 0.69 (mg RE/g)
	Methanol extract	28.65 ± 6.45 (mg RE/g)
Proanthocyanidins	Water extract	0.93 ± 0.10 (mg CE/g)
	Methanol extract	1.78 ± 0.27 (mg CE/g)

The values are mean ± standard deviation for triplicate experiments.

#### 3.3.2. Radical Scavenging Capacity

Radical scavenging activity using the DPPH assay was likewise found to be higher in the methanolic extract (Figure 3B), exhibiting consistently higher activity than the aqueous extract across all concentrations examined. Both extracts exhibited dose dependence with respect to radical scavenging ability, with the greatest difference in scavenging capacity occurring in the 100–200 µg/mL concentration range (46.16% *cf*. 2.12% at 100 µg/mL and 48.0% at *cf*. 4.70% at 200 µg/mL).

#### 3.3.3. Ion Reducing Power

Two inorganic assays—CUPRAC (Cupric Reducing Antioxidant Capacity) and FRAP (Ferric ion Reducing Antioxidant Power)—were also employed to assess ion reducing capacity of the extracts through the ability of polyphenols present in the extracts to reduce both copper (II) and ferric (III) ions respectively. The pattern of activity present in the ABTS and DPPH assays again emerged, with the methanolic extract showing twofold activity relative to the aqueous solution across the concentration range examined (Figure 3C,D). The ratios of absorbance values (H_2_O:MeOH) for both assays again showed dose dependence across the concentration range examined, with the relative ratio of absorbance (H_2_O:MeOH) being virtually identical, indicating good correlation between the methods as a measure of antioxidant capacity.

While it is difficult to quantitatively compare the results of the assays performed because of the varied nature of the chemical processes involved in each, a clear behavioural trend can be observed; namely that the use of a polar organic solvent (methanol) possesses a higher partition coefficient with respect to the capture of phenolic bioactives. Reducing power is also clearly dose dependent, a finding replicated by Jyothi *et al.* in analysing the reducing power, superoxide and hydroxide radical scavenging capacity of *E*. *tirucalli* extracts [6].

Correlation analysis of TPC and antioxidant capacity for the four methods employed to assess the reducing power of *E.* *tirucalli* extracts was undertaken and R^2^ values calculated (Table 5). ABTS and DPPH assays exhibited high *R*^2^ values (>0.998) for both the aqueous and methanolic extracts suggesting a strong correlation between TPC of the extracts and their antioxidant activity. Aqueous and methanol extracts analysed by the CUPRAC assay showed comparatively poor correlation to activity (0.718 and 0.766, respectively), while FRAP returned mixed results (H_2_O = 0.774, MeOH = 0.935).

**Table 5:** Correlation analysis of TPC and antioxidant capacity for each *E*. *tirucalli* extract.

Antioxidant Assay	R Square	
	Water Extract	Methanol Extract
ABTS	0.999	0.998
DPPH	0.998	0.998
CUPRAC	0.718	0.765
FRAP	0.774	0.935

ABTS: (2,2-azino-bis (3-ethyl-benzothiazoline-6-sulfonic acid); DPPH: (2,2-diphenyl-1-picrylhydrazyl); CUPRAC: cupric reducing antioxidant capacity; FRAP: ferric reducing antioxidant power.

### 3.4. In Vitro Assessment of Growth Inhibition Capacity of E. tirucalli Extracts

**Figure 4:**
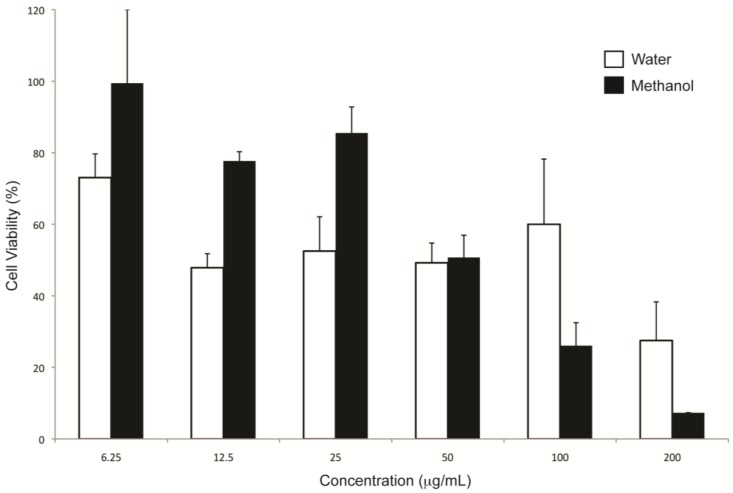
Cell growth inhibition of water and methanol *E. tirucalli* crude extracts on the MiaPaCa-2 pancreatic cancer cell line.

Cell viability assays were conducted on the pancreatic cancer primary tumour cell line (MiaPaCa2) to assess the relative toxicity of the *E. tirucalli* extracts. The toxicity of both extracts was found to be dose dependent, with cell viability decreasing with increasing extract concentration (Figure 4). Both aqueous and methanol extracts demonstrated similar activity at 50 µg/ml with a viability of ~50%, while only the methanol extract exerted a significant decrease in cell viability from 25 µg/ml. As expected, at the highest concentration of 200 µg/ml cell viability was seen to drop significantly for cells treated with the aqueous (~25%) and methanol (~7%) extracts. This trend is supported by previous studies demonstrating a link between samples with high antioxidant activity and anti-cancer properties [20,21], as well as evidence to suggest that compounds from euphorbia species may possess anti-cancer properties [1,22].

We are presently conducting further investigations to confirm the source of activity within the *E. tirucalli* extracts and to elucidate the associated cellular mechanistic pathways affected by these compounds.

## 4. Conclusions

Optimal aqueous extraction conditions for maximum yield of TPC from the leaf and stem of *E. tirucalli* were determined by response surface modelling to be 90 °C, 27 min and a plant-to-water ratio of 1:100 g/mL. TPC, flavonoid and proanthocyanidin levels in aqueous and methanol extracts were quantified using a range of analytical methods. Antioxidant and radical scavenging ability of the extracts was found to be dose dependent, with the methanolic extracts possessing the highest concentration of active constituents (MeOH > two-fold *cf*. H_2_O extracts). The extracts were found to inhibit the proliferation of pancreatic cancer cells, suggesting that the extracts may have potential to be further developed into a therapeutic treatments. Future study is recommended to further purify and examine individual bioactive compounds from these extracts and evaluate their anti-pancreatic cancer activity.
